# Otomycosis risk after non-suppurative middle ear surgery

**DOI:** 10.1016/j.bjorl.2024.101552

**Published:** 2025-01-03

**Authors:** Fatih Gul, Ozgenur Kocak, Ali Ozturk, Mehmet Ali Babademez

**Affiliations:** a Ankara Yıldırım Beyazıt University Faculty of Medicine, Department of Otorhinolaryngology, Ankara, Turkey; b Caycuma State Hospital, Department of Otorhinolaryngology, Zonguldak, Turkey

**Keywords:** Otomycosis, External acoustic canal, Tympanoplasty

## Abstract

**Objectives:**

The aim of this study was to investigate the risk factors that may cause postoperative otomycosis in patients undergoing Chronic Nonsuppurative Otitis Media (CNSOM) surgery.

**Methods:**

In this retrospective study, 409 out of 523 patients met the inclusion criteria. 44 patients diagnosed with otomycosis CNSOM were analyzed. Perioperative factors were analyzed to determine the potential risks of otomycosis. The primary factors identified as contributing to otomycosis were firmly adherent cerumen, tympano-meatal flap positioning, and Diabetes Mellitus (DM). The study analyzed the patients’ follow-up six months after the surgical procedure. Otomycosis occurring within 30-days of surgery was classified as “early otomycosis”, while those occurring later were classified as “late otomycosis”.

**Results:**

Graft success at 6-months showed no significant difference between otomycosis and non-otomycosis groups. We found that the presence of DM, tympano-meatal flap positioning, and firmly adhered cerumen removal were significantly associated with the development of otomycosis. The logistic regression model was significant and explained 9.2% of the variation. Overall, individuals with the presence of advancement flap, DM, and cerumen removal status were respectively 2.0, 2.8, and 2.1 times more likely to have otomycosis. DM was the only risk factor identified in all three patients who developed late otomycosis.

**Conclusion:**

This study found that compromised epithelial integrity in the external auditory canal, non-epithelial areas, and reduced blood circulation were independent risk factors for postoperative otomycosis.

**Level of evidence:**

4.

## Highlights

• Tympanomeatal flap advancement increases otomycosis risk by creating epithelial gaps.
• Adhered cerumen removal damages the epithelial barrier, raising otomycosis risk.
• Otomycosis does not impact tympanoplasty graft success.

## Introduction

Otomycosis, a fungal infection of the external auditory canal, is commonly influenced by factors such as individual hygiene, immunocompromised status, and humidity. Postoperative otomycosis is a distinct entity that occurs when the integrity of the external auditory canal is disrupted, as is often the case in radical mastoidectomy.

Only a few studies have investigated the frequency, rate, and association of otorrhea with postoperative conditions, and most of this research has focused on otorrhea following surgery for Chronic Suppurative Otitis Media (CSOM). In a previous study involving 1,754 CSOM patients, 4.9% of individuals experienced postoperative otorrhea, while fungal growth was observed in only 0.1% of cases.^1^ The prevalence of postoperative otomycosis varies, with studies reporting rates ranging from 0.1% to 52.8%.^2^, ^3^, ^4^ Postoperative otomycosis risk after dry middle ear surgery has not been specifically addressed in the literature.

This study aims to identify factors contributing to postoperative otomycosis, filling a gap in current literature that lacks comprehensive investigations into the risk factors associated with this complication. By exploring the interplay between otomycosis and post-surgery recovery, we contribute to a deeper understanding of the broader impact of fungal infections on surgical outcomes.

## Methods

### Study design

A total of 523 patients who had undergone Chronic Non-Suppurative Otitis Media (CNSOM) surgery was identified through retrospective chart reviews conducted between March 2019 and January 2023 at a tertiary referral hospital. Age, gender, smoking history, Body Mass Index (BMI), presence of Diabetes Mellitus (DM), previous surgeries, preoperative ear status, ear discharge, surgical notes, postoperative care were documented.

### Patient selection

The study recruited participants who had undergone a CNSOM surgery, with a specific focus on individuals who were presented with dry ears in the preoperative period. The patients who had undergone canal wall down mastoidectomy, had preoperative wet ears, had a previous ear surgery (revision), demonstrated the presence of cholesteatoma, had experienced recent ear discharge within 3-months before surgery, were using hearing aids or were lost to follow-up were excluded. Patients who had otomycosis were placed in the ‘otomycosis group’, while those without otomycosis were placed in the ‘non-otomycosis group’.

### Preoperative factors influencing postoperative otomycosis

Prior to the surgery, firmly adhered cerumen in the external ear canal was removed through aspiration or ear curette without the application of irrigation or cerumen dissolving agents. Patients were categorized into two groups: ear canal with removed firmly adhered cerumen (cerumen group) or ear canal without removed cerumen (non-cerumen group).

### Surgical procedure of external ear

In accordance with existing literature, CNSOM surgeries were performed. Postauricular sulcus incision was used to reach the middle ear. The external ear canal was incised and a tympano-meatal flap was elevated in all patients. Cartilage grafts were used in the repair of all types of tympanic membrane perforations. After the middle ear surgery, the tympano-meatal flap was either positioned in its anatomical position or advanced on the graft and secured with additional dry gel foam. A mesh soaked in antibiotic ointment was applied to the distal part of the external ear canal. These procedures were performed by experienced otologists themselves or under their direct supervision.

The rotational inferior base posterior canal skin flap tympanoplasty technique, used in patients with subtotal perforated tympanic membrane, is a simple, fast, safe, and effective method for repair.^4^ The procedures performed on the external ear canal were classified into two categories: the original position, where the external ear canal epithelium was restored to its initial location, and the advanced position, involving epithelial advancement onto the graft due to posterior marginal or total perforation of the tympanic membrane, which resulted in an epithelial gap in the external ear.

### Postoperative follow-up and care regimen

The mesh infused with antibiotic ointment was removed after a week. Then the sponge particles were aspirated, typically in the second week. Lastly, a follow-up evaluation was conducted during the third and fourth week following the procedure. The minimum follow-up was 6-months.

The diagnosis of otomycosis involved a microscopic examination of the ear to identify whitish, cottonlike fungal hyphae and/or blackish greasy spores, which was confirmed through microbiological culture. Terbinafine hydrochloride spray treatment was initiated for a duration of one week. Otomycosis cases were categorized as ‘early otomycosis’ if they occurred within 30-days after surgery, and as ‘late otomycosis’ if they occurred beyond that timeframe.

### Statistical assessment

Statistical analysis was performed using SPSS software (version 27.0). The age and BMI were assessed using the student *t*-test. The Chi-Square test was used to measure the association between two categorical variables. The logistic regression test was used to evaluate the association between a categorical variable and multiple independent variables. A *p*-value of < 0.05 was considered statistically significant.

## Results

### Patient demographics and clinical characteristics

**Fig. 1:**
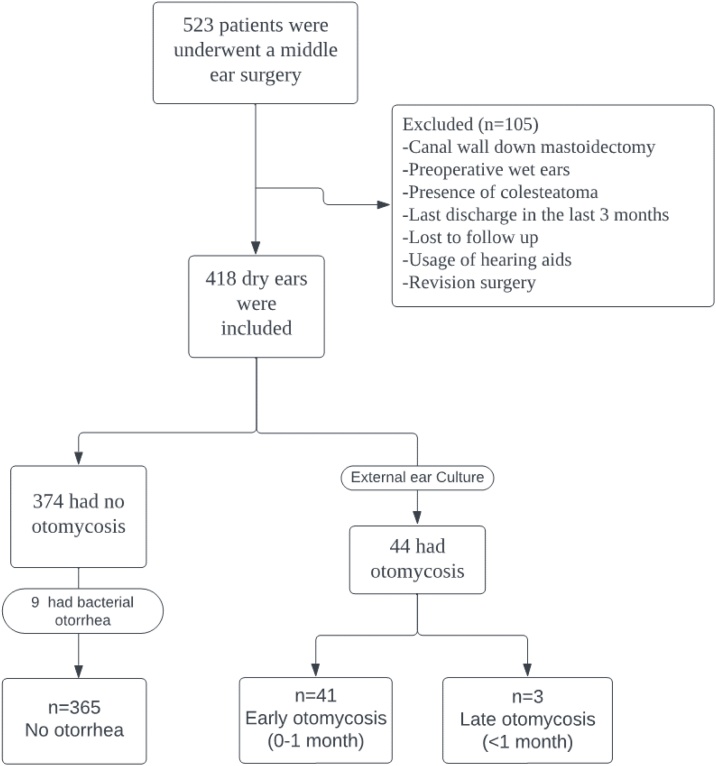
Flow diagram of the study.

Out of the initial cohort of 523 patients, 418 were included in the study based on their meeting the inclusion criteria. 9 of 418 had bacterial otorrhea. 365 participants did not present any findings of otomycosis and otorrhea. Conversely, the presence of otomycosis was diagnosed clinically and confirmed through microbial culture in 44 patients after surgical intervention (Fig. 1). Statistical analysis revealed no significant differences between the groups in terms of age, sex, BMI, or smoking status. The graft success rate was not statistically significantly different between the otomycosis group and the non-otomycosis group at 6-month follow-up (91% vs. 90%, *p* = 0.81) (Table 1).

**Table 1:** Clinical findings of the otomycosis and non-otomycosis groups.

	Otomycosis (n = 44)		Non-otomycosis (n = 365)		*p*
	n (%)	Mean	n (%)	Mean	
Age, years	‒	38.36 ± 14.07	‒	41.24 ± 18.81	0.16^a^
BMI, kg/m^2^	‒	22.65 ± 3.13	‒	23.79 ± 2.98	0.27^a^
Presence of smoking	7/37 (15%)	‒	61/304 (16%)	‒	0.32^b^
Graft success	40/44 (91%)	‒	328/365 (90%)	‒	0.81^b^

a Student *t*-test was used for numerica data.

b Chi-Square test was used for categoric data.

### Preoperative factors

The study focused on patients with dry ears in the preoperative period. Firmly adhered cerumen removal from the external ear canal was performed in 88 of 409 cases (21.5%) through curette and/or aspiration. This included 17 of 44 patients (38.6%) with otomycosis. A Chi-Square analysis revealed a significant elevation in the otomycosis group (χ^2^(1) = 8.55, *p* = 0.003). According to DM, the results of the Chi-Square test showed that there is a statistically significant association between otomycosis and the presence of DM (χ^2^(1) = 14.31, *p* < 0.001) (Table 2). Moreover, DM was the only risk factor that was identified in all three patients who developed late otomycosis.

**Table 2:** Analyses of the risk factors for otomycosis.

		Chi-square value	Otomycosis (n = 44)			Non-otomycosis (n = 365)			*p*^a^
			n	%	Adjusted Residual	n	%	Adjusted Residual	
External ear skin flap									
	Orginal position	9.206	24	7.9%	−3.0	277	92.1%	3.0	0.002
	Advanced position		20	18.5%	3.0	88	81.5%	−3.0	
Firmly adhered cerumen									
	Cerumen	8.558	17	19.3%	2.9	71	80.7%	−2.9	0.003
	Non-cerumen		27	8.4%	−2.9	294	91.6%	2.9	
Diabetes mellitus									
	Yes	14.318	9	32.1%	3.8	19	67.2%	−3.8	<0.001
	No		35	9.1%	−3.8	346	90.9%	3.8	

a Chi-Square test was used for analysis.

### Surgical status of external ear skin

Skin of the external ear canal was categorized based on epithelial restoration or advancement onto the graft. A statistically significant difference in the incidence of otomycosis was found between the groups of advancement and original position in the postoperative period (45.5% vs. 54.5%, χ^2^(1) = 9.2, *p* = 0.002) (Table 2).

### Postoperative status

Aspergillus species, with Aspergillus Niger being the most prevalent, were the primary causative agents of otomycosis, collectively responsible for 63.6% of cases (28 patients). Candida species, particularly Candida albicans, were the second most common causative agents, accounting for 26.9% of cases (16 patients). No additional fungal agents were identified. The use of terbinafine spray in the treatment of otomycosis resulted in clinical improvement in all patients, obviating the need for further culture sampling.

### Analyzing multiple factors

Further statistical analysis was conducted to identify significant associations between preoperative factors, surgical procedures, and the occurrence of otomycosis. The logistic regression test was used to evaluate the association between otomycosis and the following independent variables: cerumen removal, DM, and position of the tympano-meatal flap. Accordingly, the created model was significant and explained 9.2% of the variation. The logistic regression model was statistically significant (*p* < 0.05). Overall, advancement flap, presence of DM, and firmly adhered cerumen removal demonstrated statistically significant positive associations with the presence of otomycosis (respectively, *p* = 0.048, *p* = 0.030, *p* = 0.028). This suggests that individuals with the presence of advancement flap, DM, and cerumen removal status were respectively 2.003, 2.838, and 2.163 times more likely to have otomycosis (Table 3).

**Table 3:** Identifying significant predictor variables by using logistic regression analysis.

	Coefficient B	Standart error	*p*	Odds ratio	95% Confidence Interval
External ear skin flap	0.695	0.352	0.048	2.003	1.005‒3.991
Firmly adhered cerumen	0.772	0.351	0.028	2.163	1.088‒4.301
Diabetes mellitus	1.043	0.480	0.030	2.838	1.108‒7.274

## Discussion

The development of otomycosis after surgery is a condition that is often overlooked by otologists. However, it can prolong the healing process and require additional treatment. The literature on this topic is limited, so our study aimed to investigate the factors that contribute to the development of postoperative otomycosis.

### The identification of the factors

The literature has demonstrated that epithelial migration proceeds centrifugally, originating from the umbo of the tympanic membrane and extending laterally up to the bony cartilaginous junction at a rate of 0.05 mm per day.^5^ In cases where the external auditory canal flap is not returned to its place and needs to be advanced over the tympanic graft, the healing time is prolonged in proportion to the size of the unfilled gap. Thus, the development of epithelial gaps in the external auditory canal can make the host more vulnerable to infectious conditions during the time between when the gaps occur and when they close.

Bonding and Ravn conducted a study comparing normal ears to those who had undergone surgery due to cholesteatoma of the external auditory canal. The study found that the epithelial migration pattern and speed were normal in the healed skin; however, migration ceased when ink was applied to the crust of the ears prior to treatment.^5^ A previous study has demonstrated the presence of epithelial migration in almost all the normal ears except in 1 ear, where the migration of ink halted because of peeling of the outer epithelium of the tympanic membrane.^6^ Due to the limited applicability of softening drops in perforated ears, specialists are usually compelled to clean the firmly adhesive cerumen in the bony part of the external auditory canal using aspiration and curettage, which may potentially result in the peeling of the outer epithelium.

It has been postulated that epithelial migration is regulated by the blood vessels that provide nourishment to the epidermal layer of the tympanic membrane and the skin of the external ear.^6^ In our study, we hypothesized that inadequate blood supply may hinder epithelial migration, potentially leading to the development of otomycosis in individuals with DM. Additionally, the exclusion reason for revision surgeries is that subepithelial fibrosis, damaged blood supply and atrophy of ceruminous glands may result from repeated traumatic interventions in the external auditory canal.

### The role of cerumen removing on otomycosis

Intact skin barrier, sweat and cerumen, bacterial flora help protect the keratinized layers of the skin and create an acidic environment that inhibits fungal growth.^7^ Cerumen, rich in lysosomes, glycoproteins, antibodies, fats, and essential minerals, possesses antibacterial properties that actively contribute to safeguarding the ear’s local defense system.^8^ This fat layer plays an important role in suppressing the growth of both bacteria and fungi.^3^, ^9^ Additionally, the migration of skin in the external ear canal from medial to lateral direction helps in the removal of secretions, damaged epithelium, as well as bacteria and fungi.^10^ In short, the integrity of the skin barrier is an important factor in the prevention of fungal infections. Firmly adhered cerumen removal can damage the epithelial barrier of the external ear canal, making it more susceptible to fungal infection. In our study, the risk of otomycosis was more than twice as high in patients who had cerumen removed than in those who did not.

### The role of epithelial gap on otomycosis

The integrity of the bone and skin of the external auditory canal appears to be important factors in the development of otomycosis. The literature indicates that previous otological procedures, especially those that result in mastoid cavity, may be a potential risk factor for otomycosis.^11^, ^12^ In another study, the development of recurrence and residual risk of otomycosis was associated with the mastoid cavity and otomycosis was found to be more common in revision surgeries.^13^ Patients who underwent radical mastoidectomy and revision surgery were not included in our study in order not to affect the results in the postoperative period.

The tympano-meatal advancement flap, a flap of bony skin of the external ear, is used to cover the tympanic membrane during surgery.^14^ The finding that the position of the tympano-meatal flap may be a risk factor for otomycosis is novel. In our study, the risk of otomycosis was higher in patients who had the tympano-meatal flap advanced onto the graft than in those who had the flap restored to its original position. This suggests that the advancement of the tympano-meatal flap may create a gap in the external ear canal, which can provide a niche for fungal growth.

### The role of DM on otomycosis

Wound healing is delayed in individuals with DM due to chronic inflammation, impaired angiogenesis, and reduced endothelial progenitor cells.^15^ Upon scrutinizing the incidence of otomycosis in our patient population with DM, we observed a higher prevalence of cases in the otomycosis group, especially in the late period. This observation may be attributable to delayed wound healing in patients with DM. It may be important to shorten the follow-up interval, be vigilant for signs and symptoms of infection, and initiate treatment promptly in patients with a history of DM.

### The role of otomycosis on graft success

Research indicates that the dry or wet state of the ear before tympanoplasty and the presence of otorrhoea during the recovery period after tympanoplasty have no effect on the success rates of graft.^1^, ^16^, ^17^ Additionally, the wet or dry state of the middle ear does not impact the long-term outcomes of tympanoplasty in non-cholesteatomatous chronic otitis media.^18^ To assess the impact of otomycosis on graft success following tympanoplasty, we excluded cases with purulent otorrhea and examined the presence of otomycosis during the post-tympanoplasty recovery period. Our study provides evidence that otomycosis does not appear to affect the success of tympanoplasty grafts. This finding is consistent with the literature, which has shown that otorrhea does not affect graft success after tympanoplasty.

### Consideration of additional risk factors

A comprehensive review of the literature on risk factors for otomycosis reveals several factors contributing to fungal infections in the external auditory canal, particularly those that compromise the skin’s mechanical barrier.^19^ Frequent headphone use, scratching with rigid objects, and skin injuries are common causes of such disruptions.^20^ Additionally, disturbances in the external ear canal microbiota and reduced cerumen production can weaken natural defenses.^21^ The overuse of chemicals like soaps, shampoos, boric acid, and antiseptics, as well as excessive antibacterial ear drops, can disrupt the microbial balance, leading to fungal overgrowth.^22^ Alternative treatments such as coconut oil or mustard oil have also been linked to an increased risk of otomycosis.^23^ Activities like swimming, sauna use, and spa treatments can further predispose individuals to infection by increasing moisture exposure. Certain medical conditions and treatments heighten the risk, including DM, immunosuppressive therapy, prolonged antibiotic use, retroviral infections, sinonasal or nasopharyngeal malignancies, and a history of ear surgeries.^19^ Patients with the presence of a mastoid cavity are also at higher risk.^24^ Understanding these additional risk factors provides a broader perspective on the causes of external ear canal fungal infections and underscores the importance of considering these variables in clinical evaluations and future research.

Although several potential risk factors for otomycosis were not included in our study, this does not negate their relevance. Additionally, while the associations between the identified factors and otomycosis were statistically significant, it is important to recognize that correlation does not necessarily indicate causation. Our decision to focus solely on surgical intervention as a risk factor for otomycosis was made due to the difficulty in standardizing that variables. The exclusion of that variables, due to the retrospective nature of the study, limits the ability to capture and standardize factors, offering an incomplete view of otomycosis risk.

### Limitation

This study’s limitations include the use of oral antibiotics for dry ear, which, while aimed at preventing surgical site infections, may contradict recommendations from otolaryngological authorities. However, no evidence links oral amoxicillin and clavulanic acid to otomycosis development. Additionally, as a retrospective study, it is subject to inherent biases, including selection bias and variability in data quality. The absence of a comprehensive control group further limits the ability to draw causal inferences between surgical interventions and the risk of otomycosis. Moreover, differences in surgical techniques and surgeons' expertise were not considered, and the specific effects of patient factors such as age, BMI, and diabetes were not thoroughly analyzed. Future prospective studies with larger and more diverse control groups, as well as detailed stratification by surgical technique and surgeon expertise, are necessary to confirm and expand upon these findings.

## Conclusion

The purpose of this study was to identify potential factors that could contribute to the occurrence of otomycosis in patients who have undergone a CNSOM surgery, and to alert healthcare professionals to the issue of otomycosis when these factors are present. Possible factors that could increase the risk of otomycosis include compromised epithelial integrity in the external auditory canal, non-epithelial areas, and reduced blood circulation. If there are predisposing factors present, it may be necessary to shorten the follow-up interval and conduct extensive mycological examinations to determine the most effective treatment.

## Informed consent

Informed consent was obtained from all individual participants included in the study.

## Ethical approval

All procedures performed in the studies involving human participants were in accordance with the ethical standards of the institutional and/or national research committee and with the 1964 Helsinki declaration and its later amendments or comparable ethical standards.

## Funding

This research received no specific grant from any funding agency in the public, commercial, or not-for-profit sectors.

## Declaration of competing interest

The authors declare no conflicts of interest.
